# A Tetracycline-Repressible Transactivator System to Study Essential Genes in Malaria Parasites

**DOI:** 10.1016/j.chom.2012.10.016

**Published:** 2012-12-13

**Authors:** Paco Pino, Sarah Sebastian, EunBin Arin Kim, Erin Bush, Mathieu Brochet, Katrin Volkmann, Elyse Kozlowski, Manuel Llinás, Oliver Billker, Dominique Soldati-Favre

**Affiliations:** 1 Department of Microbiology and Molecular Medicine, CMU, University of Geneva, 1 Rue Michel-Servet, 1211 Geneva 4, Switzerland; 2 Wellcome Trust Sanger Institute, Hinxton, Cambridge CB10 1SA, UK; 3 Department of Molecular Biology and Lewis-Sigler Institute for Integrative Genomics, 246 Carl Icahn Lab, Princeton University, Princeton, NJ 08544, USA

## Abstract

A major obstacle in analyzing gene function in apicomplexan parasites is the absence of a practical regulatable expression system. Here, we identified functional transcriptional activation domains within Apicomplexan AP2 (ApiAP2) family transcription factors. These ApiAP2 transactivation domains were validated in blood-, liver-, and mosquito-stage parasites and used to create a robust conditional expression system for stage-specific, tetracycline-dependent gene regulation in *Toxoplasma gondii*, *Plasmodium berghei*, and *Plasmodium falciparum*. To demonstrate the utility of this system, we created conditional knockdowns of two essential *P. berghei* genes: profilin (PRF), a protein implicated in parasite invasion, and N-myristoyltransferase (NMT), which catalyzes protein acylation. Tetracycline-induced repression of PRF and NMT expression resulted in a dramatic reduction in parasite viability. This efficient regulatable system will allow for the functional characterization of essential proteins that are found in these important parasites.

## Highlights

► Transactivating regions within Apicomplexan AP2 family transcription factors identified ► Design of a TetRep-inducible system based on ApiAP2-derived activating domains ► Conditional disruption of the essential *profilin* gene validates the inducible system ► Depletion of N-myristoyltransferase activity severely hampers parasite development

## Introduction

Access to a generalizable system that tightly controls gene expression has been long sought after in molecular malaria research. In other parasitic organisms, such as *Trypanosoma brucei*, RNA interference (RNAi) is widely used in combination with a tight tetracycline repressor (TetRep)-based system (Bastin et al., 2000; Wirtz and Clayton, 1995). However, the application of RNAi-based regulation in the *Plasmodium* species is not broadly applicable (Mohmmed et al., 2003), presumably due to the absence of a complete RNAi machinery (Baum et al., 2009). An alternative strategy based on the FLP recognition target (FRT)-specific/FLP recombinase system has been demonstrated to work in *Plasmodium* (O’Neill et al., 2011; van Schaijk et al., 2010), and this system despite its irreversibility, offers the ability to investigate gene essentiality at restricted developmental stages (Combe et al., 2009). More recently, the control of protein stability with destabilization fusion domains has been effectively applied to some *P. falciparum* genes (Armstrong and Goldberg, 2007; Dvorin et al., 2010; Russo et al., 2009).

In the related apicomplexan parasite *Toxoplasma gondii*, an inducible gene expression system was established based on a transactivator composed of the TetRep fused to a nonendogenous activating domain (TATi1) (Meissner et al., 2002). Despite its artificial nature (being derived from a normally noncoding sequence of the plasmid backbone), this element isolated from a genetic screen in the parasite has proven to be extremely valuable to dissect the mechanistic function of numerous essential *T. gondii* genes, such as those implicated in invasion (Buguliskis et al., 2010; Huynh and Carruthers, 2006; Meissner et al., 2002; Plattner et al., 2008). The transactivator binds via the TetRep to *tet* operator sequences (*TetO*) placed in front of a minimal promoter and activates transcription. In the presence of tetracycline or the derivative anhydrotetracycline (ATc), the affinity of the TetRep for the *TetO* is dramatically reduced and transcription is turned off (Bujard, 1999; Meissner et al., 2001). However, the adaptation of TATi-2 to *Plasmodium,* although successful in tightly controlling transgene expression from multicopy episomal plasmids (Meissner et al., 2005), failed to find utility for the construction of conditional knockouts both in *P. falciparum* and *P. berghei*, probably due to insufficient transactivation activity (three attempts to conditionally KO *Pbprf* failed with TATi2, whereas all the attempts with the transactivators identified in this study were successful; data not shown). We reasoned that a transactivation system for *Plasmodium* spp. could best be designed by relying on the parasite’s own machinery for transcriptional activation.

Despite extensive bioinformatic analyses focusing mainly on sequence similarity to known eukaryotic transcription factors, the phylum Apicomplexa has presented a paucity of transcription factors (Balaji et al., 2005; Bischoff and Vaquero, 2010; Callebaut et al., 2005). Recently, however, a group of conserved proteins containing putative Apetala2 DNA-binding domains, now known as the Apicomplexan Apetala2 (ApiAP2) protein family, have been described (Balaji et al., 2005). The *P. falciparum* genome encodes 27 ApiAP2 proteins, some of which function as specific transcription factors to regulate progression throughout the parasite life cycle (Painter et al., 2011). Each ApiAP2 protein contains between one and four copies of the signature 60 amino acid AP2 DNA binding domain, and, collectively, several studies have characterized the DNA binding specificities for the *P. falciparum* ApiAP2 family members (Campbell et al., 2010; De Silva et al., 2008; Flueck et al., 2010; Lindner et al., 2010; Llinás et al., 2008). In *P. berghei*, the ApiAP2 transcriptional regulators AP2-O and AP2-Sp have been functionally analyzed in the nonerythrocytic ookinete and sporozoite life stages, respectively, and were found to serve as master regulators of parasite development in the mosquito (Yuda et al., 2010; Yuda et al., 2009). However, no functional domains have been recognized outside of the DNA binding domain for any ApiAP2 protein to date.

## Results and Discussion

### Identifying ApiAP2 Protein Fragments that Activate Transcription

**Figure 1:**
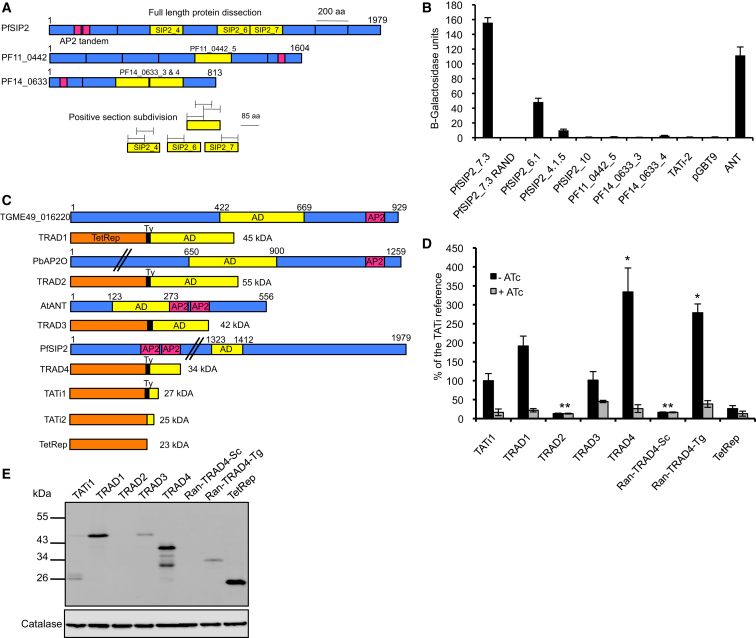
Identifying ApiAP2 Protein Fragments that Activate Transcription (A) Strategy to functionally map AP2 activation domains in yeast. DNA binding domains (AP2 motif) are shown in purple; transactivation domains (ADs) can only be identified functionally. Full-length PFF0200c/PfSIP2, PF11_0442, and PF14_0633 were dissected into sections of ∼200 amino acids (aa) shown in blue or yellow. Yellow sections tested positive for activation by colony-lift filter assay (see Table S1 for results). (B) A liquid colorimetric assay quantifying β-galactosidase in transiently transfected yeast was used to narrow down transactivation domains further. The empty cloning plasmid (pGBT9) and a section of PFSIP2 (PfSIP2_10) were used as negative controls and were negative in filter assay. AtANT was used as positive control. Error bars show standard deviations of three technical replicates. (C) Schematic representations of the TetRep-AD fusion proteins TRAD1–TRAD4 and of the full-length ApiAP2 proteins from which their ADs were derived. Putative ADs are shown in yellow, AP2 domains in purple, TetRep in orange, and Ty epitope tags in black. Expected molecular sizes are noted. (D) β-galactosidase activity in the *TetO7*-LacZ strain of *T. gondii* transiently transfected with TRAD expression constructs. Cells were grown for 48 hr in the presence or absence of ATc prior to quantification of LacZ expression with CPRG, as described (Meissner et al., 2001). The vertical axis reports β-galactosidase activity as a percentage of TATi-1-driven activity in the absence of ATc. ^∗^A synthetic version by remodeling the codons (≈50% AT) has been generated for expression in *T. gondii*. ^∗∗^The endogenous AT-rich *Plasmodium* sequence is not expressed in *T. gondii*. Error bars show the SD of three replicates from four independent experiments. (E) Western blot showing the expression levels of the different TRADs, relative to PRF in *T. gondii*. TRAD2 and ran-TRAD4-Sc are not expressed due to the AT rich codons. Note that the synthetic version of ran-TRAD4-Sc runs at the expected size when TRAD4 run aberrantly at 43 kDa. See also Figure S1.

To identify transactivating regions within ApiAP2 proteins, we used the genetically tractable models yeast and *T. gondii* to more rapidly screen for active protein sequences. First, with a one-hybrid approach in *Saccharomyces cerevisiae* (Fields and Song, 1989), three ApiAP2 proteins from *P. falciparum*, PFF0200c/PfSIP2, PF11_0442, and PF14_0633 (De Silva et al., 2008; Flueck et al., 2010; Yuda et al., 2010; Yuda et al., 2009), were dissected into overlapping sections of ∼200 amino acids (Figure 1A), each fused to the C terminus of the GAL4 DNA-binding domain (GAL4-DBD), to test for transactivation. As a positive control, we used Aintegumenta (ANT), a well-characterized plant AP2 transcriptional activator involved in the floral development of *Arabidopsis thaliana* (Krizek and Sulli, 2006). The fusions were first tested for *lacZ* expression with a yeast colony-lift filter assay (data not shown). Activating domains (ADs) found to be functional were then assessed quantitatively with a liquid β-galactosidase assay (Figure 1B and Figure S1 available online). Functional sections were further divided into shorter, overlapping peptides and examined again. Among the eleven constructs tested in the liquid assay, a peptide comprising 89 amino acids from the seventh section of PfSIP2, termed PfSIP2_7.3, was the strongest activator, while PfSIP2_6.1 and PfSIP2_4.1.5 were also clearly above background (Figure 1B). To determine whether transactivation in yeast was dependent on the PfSIP2_7.3 AD sequence or on the overall amino acid composition, we tested a randomized peptide with scrambled amino acids (PfSIP2_7.3 RAND), and it showed no activity (Figures 1B and S1B). Sequences derived from PF11_0442 and PF14_0633 subdivisions did not strongly transactivate in yeast (Figure 1B).

Recognizing that the evolutionary distance of yeast could make it a suboptimal model to identify transactivation sequences for *Plasmodium*, we turned to *T. gondii* to test a panel of candidate sequences. Gene fusions between TetRep and putative ADs (TRADs, Figure 1C) were created with a conserved domain found in TGME49_016220 (TRAD1) and PbAP2O (TRAD2)—both structural orthologs of PF11_0442 (Figure S1C)—that was inactive in the yeast assay. We also tested the AD from ANT (TRAD3) and the PfSIP2_7.3 fragment (TRAD4), which were both active in the yeast assay, and assessed the expression of LacZ under the control of a tet transactivator-responsive promoter in *T. gondii* transiently transfected with the different TRAD constructs (Meissner et al., 2002). Transactivation and inducibility were determined by X-Gal staining (Figure S1D) and compared quantitatively with a colorimetric assay (Figure 1D). All TRADs transactivated similarly or better than the reference transactivator TATi-1 (Meissner et al., 2002), with the exception of the TRAD2 construct containing the AT-rich sequence of *PbAP2O*, which was not expressed at a detectable level in *T. gondii* (Figure 1E). In contrast, the complementary DNA coding for the PfSIP2_7.3 domain in TRAD4 was codon optimized for expression in *T. gondii* and was highly active. Importantly, upon addition of ATc to the cultures, transactivation by TRAD1, TRAD3, and TRAD4 was greatly suppressed. Since the randomized PfSIP2_7.3 AD with scrambled amino acids optimized for yeast (ran-TRAD4-Sc) could not be expressed in *T. gondii* (Figure 1E), we synthesized another AD with the same sequence but optimized for expression in *T. gondii* (ran-TRAD4-Tg). Intriguingly, ran-TRAD4-Tg was both expressed and found to be as active as TRAD4 (Figure 1D), suggesting that, unlike in the yeast experiments above, amino acid composition rather than sequence may be important for transactivation by this peptide in *T. gondii*. We note that asparagine is significantly overrepresented in TRAD4, accounting for 29% of the amino acids. Previous work in yeast has demonstrated that strong transactivating sequences are rich in acidic amino acids, glutamine, proline, or asparagine, suggesting that elevated Asn may similarly play a role in *T. gondii* transcriptional activation (Ansari et al., 2001; Titz et al., 2006). However, the overall amino acid composition of other TRADs in *T. gondii* did not share striking amino acid compositional bias (Figure S1E) or predicted secondary structures (data not shown).

### Validation of the ApiAP2 Transactivators in *P. berghei* and *P. falciparum* Blood Stages

**Figure 2:**
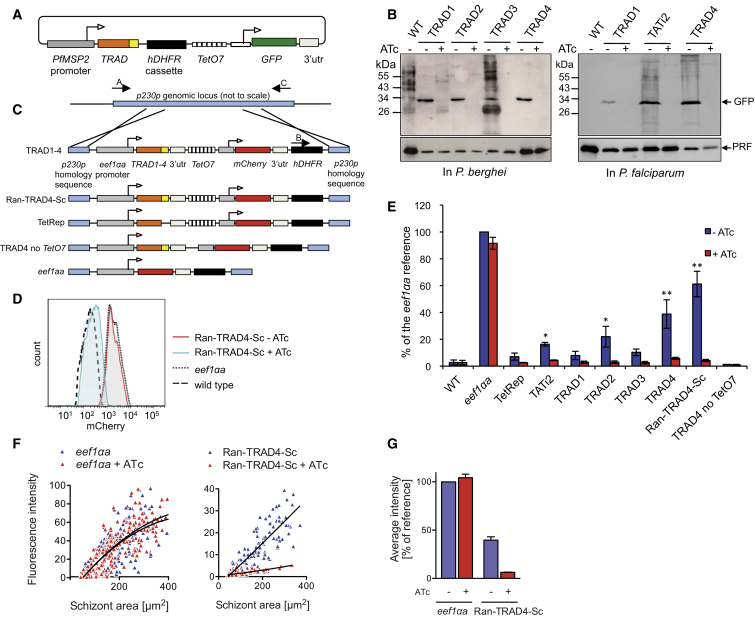
Testing of the ApiAP2 Transactivators in *Plasmodium* (A) Schematic illustrating a transfection plasmid for episomal expression in *P. berghei* and *P. falciparum*. The constructs used are derived from the previously described pMSP2-TATi2-TetO7CAM-iGFP plasmid (Meissner et al., 2005), with TATi2 replaced by the different TRADs. TRADs are under the control of a *PfMSP2* promoter, and a gpi-anchored GFP reporter is under the control of a *tet* transactivator-responsive promoter containing seven *tet* operator sites. (B) Western blot analysis of gpi-GFP expression in schizonts transiently transfected with TRAD reporter constructs as shown in (A). Infected mice were treated with ATc for 36 hr before parasites were collected and cultured into schizonts in vitro for 14 hr in the presence of 1 μg/ml ATc. *P. falciparum* synchronized cultures were treated with ATc for 48 hr and the schizonts collected. Profilin was used as loading control. Representative data from two experiments are shown. (C) Transfection vectors used to integrate TRAD expression cassettes with and *mCherry* reporter gene into the *p230p* locus by irreversible ends-out recombination. (D) Analysis by flow cytometry of the effect of ATc on mCherry fluorescence intensity in purified schizonts expressing ran-TRAD4. Wild-type schizonts and a clone carrying a stable, single-copy integration of mCherry expressed directly from the *eef1αa* promoter served as controls. Data shown are representative of three experiments. (E) Mean mCherry fluorescence intensity of schizonts carrying the constructs shown in (C) and cultured in the presence or absence of ATc. ATc was applied to infected mice and cultures as in (B). Error bars show the SD of three independent experiments. ^∗^p < 0.01 relative to TetRep. ^∗∗^p < 0.01 relative to both TetRep and TATi2. (F) Total mCherry fluorescence of individual exoerythrocytic forms (EEFs) in Hepa 1–6 mouse hepatoma cells at 50 hr p.i. is plotted against parasite area as quantified from automatically acquired images. Black lines show second-order polynomial curve fits (r^2^ = 0.64-0.75). Data are representative of two experiments. (G) Average mCherry fluorescence intensity of EEFs >100 μm^2^ is expressed as a percentage of the control line expressing mCherry under the *eef1αa* promoter without ATc. Error bars show the SD of two experiments, each measuring 50–100 EEFs for each condition.

TRADs found to be active in T. *gondii* were next assessed in malaria parasites through the use of a plasmid designed to have *tet*-regulatable transcription of GFP with the pMSP2-TRAD-TetO7CAM-gpiGFP plasmid (Figure 2A) (Meissner et al., 2005). When plasmids were maintained as stable episomes in *P. berghei*, all four TRAD constructs were expressed as functional transactivators, resulting in ATc-dependent expression of a *gpiGFP* reporter gene (Figure 2B). Using these same constructs, we tested the activity of TRAD1 and TRAD4 compared to TATi-2 in the erythrocytic stages of the human malaria parasite *P. falciparum* and found that they similarly were able to mediate ATc-dependent expression of the gpiGFP reporter (Figure 2B). Episomal plasmids are known to vary in copy number between individual parasites, which is incompatible with reproducible and homogenous cellular responses to ATc. We therefore investigated whether the TRAD fusions would still provide sufficient transactivation and control when stably integrated into the genome as a single copy gene. Transgenic parasite clones were generated with vectors designed to replace the redundant *p230p* gene (PBANKA_030600) of *P. berghei* (Janse et al., 2006a) by double homologous recombination. The resulting insertions contain a single copy of a cassette expressing different TRAD constructs under the control of the strong constitutive *eef1αa* promoter that is active throughout the life cycle of *P. berghei*, including the insect and hepatic stages (Figure 2C). In these reporter cassettes, an inducible promoter composed of seven tet operators and a minimal promoter from the calmodulin gene of *P. falciparum* controls the expression of a *mCherry* gene. Nine parasite lines with confirmed integration of different TRAD constructs were cloned by limiting dilution. Schizonts cultured in the presence or absence of ATc were analyzed for mCherry expression by flow cytometry, and the fluorescence intensity was compared to a parasite line in which the reporter was expressed directly by the *eef1αa* promoter (Figures 2D and 2E). Single copies of TATi2, TRAD1, TRAD2, TRAD3, and TRAD4 each resulted in expression of mCherry (Figure 2E). Tati2, TRAD2, and TRAD4 activated significantly stronger than TetRep without a transactivation domain. TRAD4 showed improved transactivation when compared to the previously identified TATi2 peptide. As a control, the TRAD4 peptide failed completely to transactivate in a construct lacking the *tet* operators upstream of the minimal promoter (TRAD4 with no TetO7), demonstrating that the TRADs act as genuine transactivation sequences in *P. berghei*.

Interestingly, transactivation by the sequences from *T. gondii* (TRAD1) and *A. thaliana* (TRAD3) was comparable to a *tet* repressor lacking any transactivation sequence (TetRep only), while both *Plasmodium*-derived sequences (TRAD2 and TRAD4) enhanced transactivation. As in *T. gondii*, a randomized TRAD4 peptide produced marked transactivation, which in the case of *P. berghei* was even stronger than that mediated by TRAD4 itself. In the presence of ATc, fluorescence of all TRAD reporter lines was strongly repressed to almost wild-type (WT) background levels. These data show that in *P. berghei* blood stages, TRAD2, TRAD4, and ran-TRAD4 allow different levels of transgene expression to be controlled by ATc.

### Examination of the Transactivation in *P. berghei* Mosquito and Liver Stages

We next assessed whether tet-regulatable expression would persist in the mosquito stages. On day 7 postinfection (p.i.), mCherry fluorescence intensity of ran-TRAD4-Sc oocysts reached 32% ± 3% of the *eef1αa* control parasites, suggesting that transactivation occurred. However, ATc failed to reduce fluorescence when added to the fructose solution for 3 days from day 6 p.i., during a period of rapid oocyst growth (data not shown). Similarly, ATc treatment during the 3 days preceding analysis affected mCherry fluorescence neither of day 12 oocysts nor of salivary gland sporozoites at 26 days p.i. Importantly, however, when ran-TRAD4-Sc salivary gland sporozoites were used to infect mouse hepatoma cell cultures, addition of ATc to the culture medium from 3 hr p.i. repressed mCherry expression as determined by automated microscopy and image analysis at 50 hr p.i. (Figures 2F and 2G). These data show that for the system described here to be useful in mosquito stages in vivo, either its genetic components or the delivery of ATc to the mosquito require further optimization, but that in liver stages ran-TRAD4-Sc provides an effective means of regulating expression of a transgene.

### Knockdown of Essential Genes

**Figure 3:**
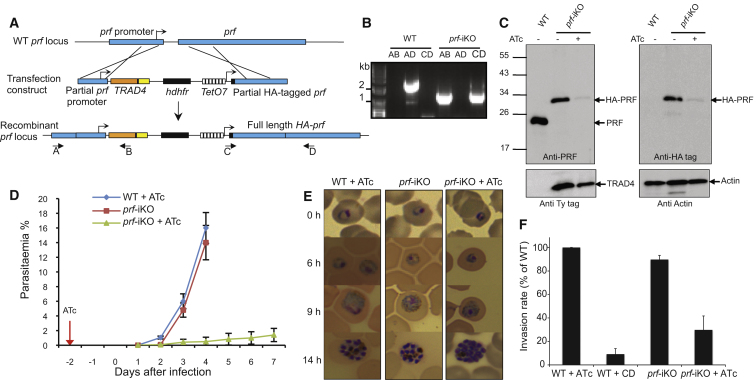
Generation of a *prf* Knockdown in *P. berghei* (A) Schematic representation of the strategy used to construct *prf*-iKO parasites. A recombination event occurs in the *prf* promoter to place the TRAD4 transactivators under the control of the *prf* promoter, the second within the *prf* coding sequence to add two N-terminal HA tags and hence placing the tagged full-length *prf* under the control of the Tet-responsive promoter. (B) PCR analysis to confirm that double homologous recombination occurred; PCRs on one representative clone are shown. (C) Western blot analysis of schizont lysates for PRF and TRAD4 expression showing the ATc-regulated expression of HA-PRF (both with anti-PfPRF and anti-HA antibodies). The inducible copy of PRF migrates slightly slower due to the double HA tag. Actin serves as loading control. The TRAD4 expression is revealed with anti-Ty antibodies. (D) Parasitaemia in mice that were treated or not with ATc in the drinking water from 2 days prior to infection. Error bars show the SD of six mice per condition. (E) Giemsa-stained blood smears showing normal development of *prf*-iKO parasites to the schizont stage in vitro. Mice were infected with WT and *prf*-iKO parasites and treated or not with ATc for 24 hr before parasites were cultured in vitro for the times indicated. Error bars show SD of three independent experiments. (F) Invasion phenotype of *prf-iKO* parasites. Vybrant Green-labeled purified schizonts were incubated with DDAO-SE-labeled RBCs, and the merozoites were allowed to invade. Parasitaemia in the DDAO-SE-labeled target population was determined by flow cytometry. Invasion efficiencies were determined as a percentage of the WT control parasites. Cytochalasin D (CD)-treated schizonts were used as a negative control. Data are represented as mean ± SD of three independent experiments. See also Figure S2.

We next asked whether a TRAD4-based ATc-repressible transactivator system could be used to conditionally knock down profilin (*prf*, PBANKA_083300) and N-myristoyltransferase (*nmt*, PBANKA_102980) genes, which are both predicted to be essential to *Plasmodium* development in asexual blood stages. Previous studies have established the importance of PRF as a regulator of actin dynamics in invasive stages of Apicomplexa (Baum et al., 2008; Kursula et al., 2008; Plattner et al., 2008). In *T. gondii*, PRF is not only essential for gliding motility, host cell invasion, and egress, but also acts as a modulator of the host immune response via TLR11 (Kursula et al., 2008; Plattner et al., 2008). A double crossing over recombination strategy was used to position *TRAD4* upstream of the endogenous *P. berghei prf* promoter, while the coding sequence of *prf* was brought under the control of the inducible promoter containing the *tet* operator. Simultaneously, two HA epitope tags were placed at the N terminus of the resulting inducible copy of *prf* (Figure 3A). Several independent transgenic pools were obtained (five different transfections were attempted, and all resulted in positive transgenics pools), cloned, and assessed by genomic PCR (Figure 3B). Expression of TRAD4 as well as the ATc-induced depletion of HA-PRF were confirmed by western blot (95% downregulation compared to WT and 90% downregulation compared to the untreated *prf*-iKO) (Figures 3C, S2A, and S2B). In mice infected with *prf*-iKO parasites in the absence of ATc, the rise in parasitaemia was indistinguishable from WT infections in the presence of ATc (Figure 3D). In contrast, an extremely low parasitaemia was detected with *prf*-iKO in the presence of ATc (Figure 3D). In addition, we observed a modification of stage composition of *prf*-iKO parasites depleted in PRF in peripheral blood (Figure S2C), with a decreased proportion of ring stages and the appearance of circulating mature segmented schizonts. While the latter points to a defect in egress, the former would also be consistent with an invasion phenotype. Consistent with this, when we exposed *prf*-iKO parasites to ATc for 24 hr in mice and then continued this treatment for 14 hr in vitro, morphologically mature schizonts were formed (Figure 3E), suggesting that intraerythrocytic development was not visibly affected.

For examination of whether *prf*-iKO parasites had an invasion defect, mature schizonts were purified, labeled, and mixed with uninfected red blood cells (RBCs) that had been treated with a fluorescent cell tracer. Schizonts were then ruptured mechanically by vigorous shaking, and merozoites were allowed to invade the labeled target RBCs in vitro. Newly invaded erythrocytes were identified and counted by fluorescence-activated cell sorting (FACS) as cells that combined the parasite DNA label with the tracer dye of the host cell (Theron et al., 2010). The number of newly invaded erythrocytes produced from the same number of morphologically mature schizonts was reduced by 60% upon ATc treatment of *prf*-iKO parasites (Figures 3F and S2D). While these data cannot distinguish between phenotypes affecting either forced egress or invasion, they confirm and explain the altered stage composition of *prf*-iKO parasites in peripheral blood in presence of ATc (Figure S2C) and suggest that PRF plays an important role at around the time *P. berghei* transitions from one erythrocyte to the next. This is consistent with the reported role for PRF during invasion and egress in *T. gondii* (Plattner et al., 2008). Knockdown of *prf* had no effect on gametocyte production (Figure S2E), and microgametocytes from treated mice released gametes effectively when stimulated in vitro (Figure S2F). When gametocytes from treated mice were stimulated to form gametes and then cultured in the continued presence of ATc, PRF levels in ookinetes (Figures S2G and S2H) were reduced and the rate at which ookinetes arose from macrogametocytes (Figure S2H) was halved, suggesting that PRF has an additional function in fertilization or ookinete development.

Taken together, these results demonstrate that (1) TRAD4 can drive *prf* expression to a level compatible with normal parasite growth, (2) depletion of HA-PRF upon ATc treatment is sufficient to reveal a clear growth phenotype in vivo, and (3) ATc treatment of ookinetes cultured in vitro can reveal phenotypes during zygote and ookinete development.

**Figure 4:**
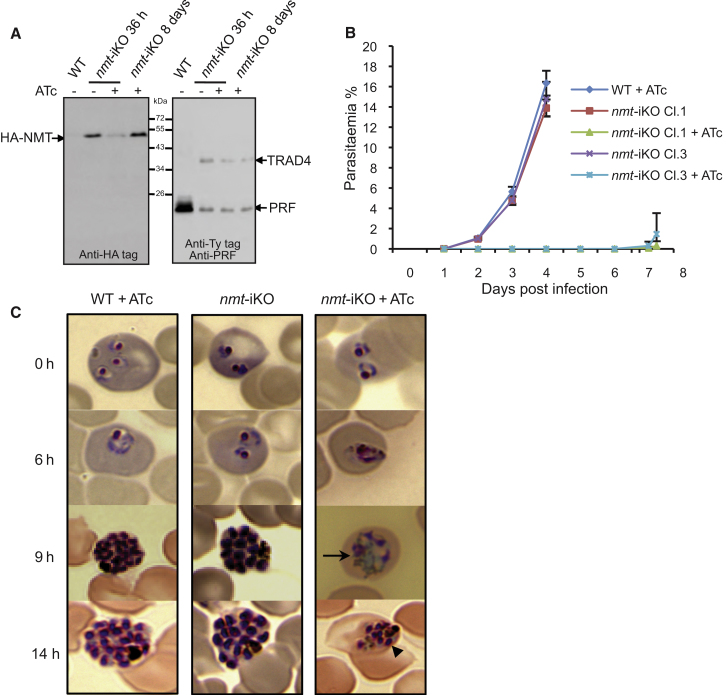
Generation of a *nmt* Knockdown in *P. berghei* (A) Western blot analysis of mixed asexual blood stages, showing that HA-NMT is expressed and initially regulated by ATc. Protein expression in parasites from mice treated with ATc for 36 hr is compared with that in ATc resistant parasites that emerged after 8 days of continuous treatment. TRAD4 is detected by anti-Ty antibodies and PRF serves as loading control. (B) Parasitaemia in mice that were treated or not with ATc in the drinking water from 2 days prior to infection. Error bars show standard deviations of 5 mice per parasite clone. (C) Giemsa-stained blood smears of in vitro matured WT and *nmt*-iKO parasites. To assess intraerythrocytic development, infected mice were treated or not with ATc for 24 hr before parasites were cultured in vitro for the times indicated. Data are representative of 3 experiments. The intra-erythrocytic development is severely perturbed and disorganized (arrow), resulting in aberrant schizonts with only a few small merozoites (arrow-head). Data are representative of 2 experiments. See also Figure S3.

The second essential gene that we targeted was the *P. berghei* *nmt*, which catalyzes the transfer of myristate from myristoyl-CoA to the amino-terminal glycine residue on numerous cellular proteins, many of which are subsequently modified by palmitoylation (Sorek et al., 2009). Myristoylation of proteins is a crucial regulatory mechanism implicated in a great variety of biological processes, and consequently NMT is essential for viability in all cell types tested to date. Notably, NMT has recently been reported as an attractive novel drug target against the protozoan parasites *T. brucei* and *Leishmania donovani* (Brannigan et al., 2010; Frearson et al., 2010), which potentially extends to the *Plasmodium* species (Bowyer et al., 2007; Rees-Channer et al., 2006). Using a similar strategy as described for *prf* above, we generated a conditional disruption of the *nmt* gene (Figure S3A). Promoter replacement and regulation by ATc were confirmed by quantitative PCR and western blot analysis of *nmt*-iKO clones (Figures 4A and S3B–S3D). In vivo development of two *nmt*-iKO clones was severely impaired in the presence of ATc. After intravenous injection of equal numbers of parasitized erythrocytes, ATc treatment of mice delayed the appearance of a detectable parasitaemia from day 2 to day 8 p.i. From day 8, only four out of ten treated mice developed a detectable and increasing parasitaemia, suggesting that a small fraction of parasites escaped the ATc treatment. By isolating these surviving parasites, we found that they had lost regulation of HA-NMT expression by ATc (Figure 4A), an in vivo phenomenon that has also been observed with the Tet system in *T. brucei.* The *nmt*-iKO parasites harvested from mice after 24 hr treatment with ATc were assayed for intraerythrocytic development in vitro. While the morphology of the ring stages collected from the mice was normal, the maturation was severely impaired, resulting in the appearance of aberrant trophozoites and schizonts (Figure 4C). Given the severity of the phenotype, we repeated the experiment by pretreating the mice with ATc for only 12 hr instead of 24 hr before the parasites were collected and taken into culture. At this point, *nmt*-iKO parasites isolated from ATc treated and untreated mice showed no difference in parasitaemia or morphology. After 14 hr of in vitro culture, the parasites that had been treated with ATc in mice for 24 hr failed to develop normally, irrespective of whether ATc was present during the subsequent culture period. In contrast, parasites exposed to ATc for only 12 hr in the mice were then able to recover and form mature schizonts in the absence of ATc in culture, whereas the continued presence of ATc resulted in a mixed population of mature schizonts and arrested/abnormal parasites.

Transcriptome data (Bozdech et al., 2003) indicates that Pbnmt expression increases during the ring stage of the erythrocytic cycle, which requires 24 hr for completion. It is plausible that after 24 hr treatment with Atc in mice, all ring stage parasites and parasites that progress through a full cycle back to ring stage have been depleted of NMT and thus fail to develop. After only 12 hr treatment, however, only parasites at ring stage are depleted, so trophozoites and early schizonts that have passed the ring stage bottleneck can develop normally in culture even in presence of ATc (Figure S3F). Approximately 50 proteins are predicted substrates for NMT in *P. falciparum* (Jones et al., 2012), so downregulation of NMT most likely affects numerous cellular functions in the parasite.

### Conclusion

The identification of highly functional transactivating domains has allowed us to develop a robust Tet-repressible transactivator system that is well suited for the study of genes essential for the red blood stages of *P. berghei* development. This regulatable system will allow the direct validation of candidate drug targets to combat malaria, as highlighted by our results with NMT. Although all proof-of-principle data for the *prf* and *nmt* genes were obtained using TRAD4 in *P. berghei*, this study provides a panel of transactivation sequences of varying strengths, which will be useful to achieve tailored levels of regulatable gene expression. Of course, optimizing the system to *P. falciparum* is another major goal. Data presented here show that for *P. falciparum*, TRAD4—and to a lesser extent TRAD1—transactivates conditionally when expressed from an episomal plasmid in the asexual blood stages. However, further work is required to investigate whether any of the TRADs identified here are superior to TATi2 when integrated as single copies into the genome of the human parasite. Another remaining challenge is to apply this system to essential genes in the mosquito and liver stages of the *Plasmodium* life cycle. While our initial analysis of a *prf-*iKO indicates that TRAD4 transactivates conditionally in ookinetes, our attempts to regulate expression of mCherry at the oocyst and sporozoite stages were unsuccessful, highlighting the need to optimize either the genetic system or the delivery of ATc to mosquitoes. For such studies, it may be necessary to rear mosquitoes on a larval diet free from tetracycline antibiotics that could interfere with the expression system when carried over into the adult mosquito. Ran-TRAD4-Sc allowed for conditional transactivation in *P. berghei* liver stages; thus, the Tet system could potentially be utilized to study gene function at this stage.

## Experimental Procedures

### Identification and Testing of *Plasmodium falciparum* ApiAP2 Protein Fragments for Their Ability to Activate Transcription in Yeast

To identify putative functional transactivating regions in the ApiAP2 family of transcription factors, we dissected the PFF0200*c* (*pfsip2*), PF14_0633, and PF11_0442 genes into regions encoding ∼200 amino acids and cloned these fragments. We focused on these three ApiAP2 genes because all three have been characterized and reported in the literature and contain functional AP2 domains that bind specific DNA sequence motifs (Campbell et al., 2010). The *P. berghei* homologs of PF11_0442 (PBANKA_090590) and PF14_0633 (PBANKA_123980) have been shown to be essential developmental regulators in the mosquito stage (Yuda et al., 2010; Yuda et al., 2009). We noted that PF11_0442 also has a region of homology outside of the AP2 domain with *Toxoplasma gondii* protein TGME49_016220 and other *Plasmodium* species. In addition, the protein alignment of these orthologs shows that the N- and C-terminal ends of PF11_0442 are not conserved in TGME49_016220. We therefore only tested the aligned regions excluding the tails present in *Plasmodium* (Table S1). Finally, PFF0200c (PfSIP2) has been demonstrated to act as a scaffolding protein to recruit additional proteins to the subtelomeric regions of *P. falciparum* chromosomes (Flueck et al., 2010). As a positive AP2 control, we used the *A. thaliana* protein Aintegumenta (ANT), because it has already shown to activate transcription in this system and the activating domain was mapped precisely (Krizek and Sulli, 2006). We also tested two synthetic transactivators, TATi-1 and TATi-2, which have previously been shown to function in *T. gondii* (Meissner et al., 2002) and in *P. falciparum* (TATi-2 only) (Meissner et al., 2005).

Protein alignments against orthologs from other apicomplexan species were used to guide our selection of fragments in order to avoid disrupting any regions of homology. PCR primers were designed to introduce restriction sites (EcoRI and SalI) at the section ends for cloning into pGBT9 (gift from Beth Krizek) (Krizek and Sulli, 2006). The final constructs were transformed into *Escherichia coli* strain DH5α and encode fusions to the C-terminal end of the GAL4 DNA binding domain (GAL4-DBD). PCR-positive clones were purified (QIAGEN) and were confirmed by DNA sequencing. Recovered plasmids were subsequently transformed into the yeast strain HF7c [MATa, ura3–52, his3–200, lys2–801, ade2–101, trp1–901, leu2–3, 112, gal4–542, gal80–538, LYS2::GAL1–HIS3, URA3::(GAL4 17mers)3–CYC1–lacZ] (gift from Beth Krizek) to test for transactivation with a one-hybrid system. Yeast competent cell transformation used a modified lithium acetate procedure (Gietz et al., 1992). Transformed cells were plated on SD/-Trp plates and grown for 3 days at 30°C. We first qualitatively assayed all of our yeast strains for activation of a *lacZ* gene reporter using a colony-lift filter assay to screen for the production of the enzyme β-galactosidase using X-gal (Breeden and Nasmyth, 1985).

### Refinement of the Activation Sequences

Since PfSIP2_4, PfSIP2_6, PfSIP2_7, and PF11_0442_5 activated transcription in a yeast colony-lift assay, we subdivided each of these into three overlapping sections of ∼85 amino acids each. The first of the subdivisions spanned the first half of the section, the third subdivision spanned the second half of the section, and the second subdivision completely overlapped the two to ensure that the activation domain was not interrupted (e.g., PfSIP2_7.1, PfSIP2_7.2, and PfSIP2_7.3; see Figure 1A). These sections were cloned into pGBT9 as described above and retested for transcriptional activation in the colony-lift assay. PfSIP2_4.1, PfSIP2_4.2, PfSIP2_6.1, and PfSIP2_7.3 all produced positive results. Since PfSIP2_4.1 and PfSIP2_4.2 were both able to activate transcription, this indicated that the region of overlap between the two sections contained the activation sequence. Therefore, a smaller 54 amino acid region (PfSIP2_4.1.5) was also tested, and it was able to activate transcription. Therefore, PfSIP2 provided us with three functional transactivating regions. In contrast, all subdivisions of PF11_0442_5 failed to activate transcription, so the section was kept whole for further testing.

For those yeast clones that performed well in the colony-lift assay, we further tested them using a quantitative liquid chlorophenolred-β-D-galactopyranoside (CPRG) assay to compare β-galactosidase activity (Miller, 1992). We also generated a randomized version for one of the functional protein segments (PFF0200c_7.3) to determine whether activation was specific to the protein sequence or overall amino acid content. To do this, we wrote a MATLAB code to randomize the input sequence (Figure S3), which we codon optimized for expression in *S. cerevisiae* (PFF0200c_7.3_RAND). This gene was synthesized by DNA2.0 and cloned into pGBT9 as described above. The following 11 constructs were tested in the liquid assay: ANT, PFF0200c_7.3, PFF0200c_7.3_RAND, PFF0200c_6.1, PFF0200c_4.1.5, PFF0200c_10, PF11_0442_5, PF14_0633_3, PF14_0633_4, TATi-2, and the plasmid pGBT9 with no insert. ANT was used as a positive control, and pGBT9, TATi-2, and PFF0200c_10 were used as a negative control.

β-galactosidase units were calculated with the formula

$$\text{β}\text{-}\text{galactosidase}\;\text{units}=\frac{1000\times \text{OD}578}{\left(\text{t}\times \text{V}\times \text{OD}600\right)},$$

where t = elapsed time (in min) of incubation, V = 0.1 × concentration factor (V = 0.5 in this study), and OD600 = A600 of 1 ml culture.

### Parasites and Mosquitoes

A *T. gondii* RH strain expressing HXGPRT and LacZ under the control of a tet transactivator-responsive promoter (Meissner et al., 2001) was used to validate the ApiAP2 transactivators in *T. gondii. T. gondii* tachyzoites were grown in human foreskin fibroblasts (HFFs) or Vero cells in Dulbecco’s Modified Eagle’s Medium (DMEM) (GIBCO BRL, http://www.invitrogen.com/) supplemented with 10% fetal calf serum (FCS), 2 mM glutamine, and 25 μg/ml gentamicin.

All *P. berghei* parasites are derivatives of clone 2.34 of the ANKA strain. Ookinetes were produced in vitro by culturing of gametocyte-infected mouse blood in ookinete medium (RPMI1640 containing 25 mM HEPES [Sigma], 10% FCS, and 100 μM xanthurenic acid [pH 7.5,] with or without ATc at 1 μg/ml) for 24 hr at 19°C, and conversion assays were performed by live staining of ookinetes and activated macrogametes with Cy3-conjugated monoclonal antibody 13.1 against the P28 surface protein. The conversion rate from macrogametes into ookinetes was determined as the number of elongate ookinetes as a percentage of the total number of Cy3-fluorescent cells.

For transmission experiments *Anopheles stephensi*, strain SD500 was allowed to feed on female Theiler’s Original (TO) outbred mice 3 days after intraperitoneal injection of infected blood. Unfed mosquitoes were removed the day after, and mosquitoes were maintained on 8% fructose/water at 19°C. Oocysts were imaged on dissected midguts 9 and 12 days after feeding with a M205FA stereomicroscope and LAS AF software equipped with a DFC340 FX monochrome camera (all from Leica). mCherry fluorescence intensity of oocysts was analyzed with the SpotDetector BioApplication image analysis software (ThermoFisher). Sporozoites isolated from infected salivary glands at 26 days p.i. were imaged with a DM2500 widefield microscope (Leica) for quantification. For in vitro sporozoite infections, Hepa 1–6 (ATCC CRL-1830) mouse hepatoma-cells were cultured in DMEM high glucose (GIBCO/Invitrogen) with 10% FCS (Tet System Approved Serum, Clontech) at 37°C with 5% CO_2_. For sporozoite infection (the protocol was adapted from Prudêncio et al. [2008]), Hepa 1–6 cells were seeded on 96-well cell culture plates (10,000 cells per well) and cultured for 24 hr. Twenty thousand *eef1αa* or ran-TRAD4-Sc isolated salivary gland sporozoites were added to the cells in DMEM, 10% FCS, and 1% pen/strep. At 3 hr p.i., the medium was exchanged for fresh medium with or without 1 μg/ml ATc and kept in the dark in the incubator. At 50 hr p.i., the cell nuclei were counterstained with Hoechst 33342 (0.2 μM, Molecular Probes/Invitrogen) in fresh medium (no ATc), and the plate was placed inside a life cell chamber (37°C with 5% CO_2_). The images of wells were automatically acquired with a Cellomics ArrayScan VTI HCS Reader (20×; 100 fields per well) and analyzed with the SpotDetector BioApplication (all from ThermoFisher) for mean fluorescence intensity of parasites.

*P. falciparum* strain 3D7 was grown in A+ erythrocytes in RPMI-1640 medium with glutamine (Life Technologies, http://www.invitrogen.com/), 0.2% sodium bicarbonate, 25 mM HEPES, 0.2% glucose, 5% human serum, and 0.1% Albumax II (Life Technologies). Parasites were synchronized by a double sorbitol treatment as previously described (Lambros and Vanderberg, 1979).

### *T. gondii* and *P. berghei* and *P. falciparum* Transfection

All animal work was conducted under a license issued by the Swiss Veterinary Office in accordance with cantonal and international guidelines. Transient transfections of *T. gondii* strain were undertaken as previously described (Soldati and Boothroyd, 1993). Stable transformants were selected for expression of hDHFR. *P. berghei* erythrocytic stages were transfected by electroporation with Amaxa technology as previously described (Janse et al., 2006b).

*P. falciparum* erythrocytic stage parasites were transfected as previously described (Crabb et al., 2004). Transfection of Plasmodium clones 3D7 were carried out by electroporation, and selection was achieved by treatment of parasites with 5 nM of WR99210 for human dhfr-based plasmids.

### *P. berghei* mCherry FACS Analysis

Schizonts cultured for 24 hr in vitro were purified with a Nycodenz cushion and stained for 15 min with Hoechst 33342 DNA dye (Invitrogen). Cells were analyzed with the 488 nm and 355 nm laser lines of a BD LSR Fortessa flow cytometer (BD Biosciences). mCherry fluorescence was detected by a 610/20 filter and Hoechst by a 450/50 filter. BD FACSDiva software (BD Biosciences) was used to collect 10,000 events for each sample. The data collected was further analyzed with FlowJo (Tree Star). The schizont population was identified by Hoechst stain, and average mCherry fluorescence was then determined in the gated schizont population.

### *P. berghei* Invasion Assay

The invasion assay was adopted from that described for *P. falciparum* by Theron et al. (2010). Schizonts cultured for 16 hr in vitro were purified with a Nycodenz cushion, and parasite DNA was stained with Vybrant Green (Invitrogen) according to the manufacturer’s recommendations. Target erythrocytes were labeled with the amine-reactive fluorescent dye DDAO-SE (Invitrogen) according to the manufacturer’s instructions. Labeled parasites and target RBCs were mixed at ratio 1/100, schizonts were then ruptured mechanically by vigorous shaking, and merozoites were allowed to invade in vitro for 20 min. As a control, schizonts were treated with cytochalasin D (CD) to inhibit invasion. Schizont rupture and invasion were confirmed on Giemsa-stained blood smears. Samples were then fixed (paraformaldehyde 2%, glutaraldehyde 0.05%) and analyzed by FACS (FACSCalibur, BD). Vybrant Green was detected by a 530/30 filter and DDAO-SE by a 661/16 filter. The data collected were further analyzed with FlowJo (Tree Star). The parental parasite population was identified by Vybrant Green stain, the target RBCs by DDAO-SE stain, and the newly invaded erythrocytes by Vybrant Green and DDAO-SE stain.

### PCR Analysis of the *prf* and *nmt* Loci

Genomic DNA isolated from clones of pyrimethamine-resistant parasites transfected or from wild-type parasites was subjected to PCR with the primers indicated (Figure 4A and Table S1).

### Infection of Mice with *P. berghei* Parasites

The blood stages of cloned lines of *P. berghei* were maintained as stabilates (parasitized erythrocytes in Alsever solution) in liquid nitrogen. Mice were infected intraperitoneally with 10^7^ parasites. Parasitaemia was determined daily by means of blood smears stained with Giemsa and a count of the number of infected erythrocytes in at least 1,000 erythrocytes.

### ATc Treatments

For in vivo treatment of mice, ATc (SIGMA) was dissolved in water + 5% sucrose at a concentration of 0.2 mg/ml. The drinking water bottle was wrapped in aluminum foil to prevent precipitation of ATc due to light and the solution changed every 48 hr. For in vitro treatments, ATc was diluted in culture medium at 1 μg/ml as previously described (Meissner et al., 2005). For tetracycline treatment of mosquitoes, ATc was dissolved freshly in the fructose feeding solution (8% fructose/water) at a concentration of 100 μg/ml and kept in the dark, and the solution was exchanged every 24–48 hr. *eef1αa*- or ran-TRAD4-Sc-infected mosquitoes were treated for 3 consecutive days before dissection. For sporozoite-infected hepatoma cultures the culture medium was exchanged at 3 hr p.i. for fresh medium with or without 1 μg/ml ATc and kept in the dark in the incubator.

## Supplemental Information

Document S1. Supplemental Experimental Procedures, Figures S1–S3, and Table S1
