# Supplementary material for: A Tetracycline-Repressible Transactivator System to Study Essential Genes in Malaria Parasites
Source: Cell Host Microbe. 2012 Dec 13;12(6):824–34. doi: 10.1016/j.chom.2012.10.016 (PMC3712325; doi:10.1016/j.chom.2012.10.016)
Supplement: Document S1. Supplemental Experimental Procedures, Figures S1–S3, and Table S1 [file mmc1.pdf]

Cell Host & Microbe, Volume *12*

Supplemental Information

A Tetracycline-Repressible Transactivator System to Study Essential Genes in Malaria Parasites

Paco Pino, Sarah Sebastian, EunBin Arin Kim, Erin Bush, Mathieu Brochet, Katrin Volkmann, Elyse Kozlowski, Manuel Llinás, Oliver Billker, and Dominique Soldati-Favre

Supplemental Experimental Procedures

Construction of Plasmids

T. gondii transfection plasmids.

All the *T. gondii* transfection plasmids derive from pTUB8-TATi1-HX (Meissner et al., 2002). cDNA coding for the various AD were amplified using total RNAs prepared from freshly lysed RH tachyzoites or *P. berghei* with a Trizol Kit (Invitrogen, http://www.invitrogen.com). TGME49_016220 AD was amplified from genomic DNA, AtANT was amplified from pGDB-AtANT (Krizek and Sulli, 2006). The final version of PfSIP2 AD is a synthetic gene (http://www.geneart.com). All ADs were cloned into pTUB8-TATi1-HX to generate pTUB8-TRAD-HX constructs.

P. berghei transfection plasmids.

The plasmids used in *P. berghei* derive from pTGPI-GFP (Meissner et al., 2005). The transactivator TATi2 was replaced by the different TRADs to generate pTRAD-GPI-GFP, for evaluation of transactivation and regulation by ATc in *P. berghei* when transfected as episomes. For stable integration of a single copy of TRAD4 into the genome a TetO7-gpiGFP expression cassette from pTGPI-GFP (Meissner et al., 2005) was inserted into plasmid p230p(DCO) , a plasmid analogous to pL0018 which allows irreversible integration into the redundant *p230p* genomic locus by ends-out recombination (Janse et al., 2006b). The *eef1αa* promoter and TRADs1-4, as well as ran-TRAD4, were then inserted to generate p230p-eef1αa-TRAD-hDHFR-TetO7-gpiGFP-p230p. Next, gpiGFP was replaced with the mCherry coding sequence. For the TetRep control, the activation domain of the TRAD2 construct was removed, and a stop codon added to the TetRep sequence. For the construct encoding TRAD4 without TetO7, the seven *tet* operator sites were removed from the TRAD4 construct, leaving only the minimal promoter upstream of the mCherry coding sequence. For comparison of mCherry expression levels we also analysed a parasite clone in which an mCherry protein of identical sequence is expressed directly under the control of the *eef1αa* promoter from within the *p230p* locus (Janse et al., 2006a).

Conditional KO constructs.

The *prf*-iKO construct was generated by replacing the GPI-GFP in the pTGPI-GFP by gDNA containing the first 3 of the 4 exons of the *prf* gene fused with 2 HA tags at the N-terminus. Then the PfMSP2 promoter driving TRAD4 expression was replace by the *prf* promoter. The TetO7HA-PRF expression cassette was removed from the plasmid and recloned at a different position to generate the final iKO construct p5'*prf*TRAD4 –hDHFR-TetO7HA$_2$PRFΔ.

The *nmt*-iKO construct was generated by replacing the *prf* coding sequence in p5'*prf*TRAD4 –hDHFR-TetO7HA$_2$PRFΔ by 650bp of gDNA coding for *nmt* fused with 2 HA tags at the N-terminus. Then the *prf* promoter driving TRAD4 expression was replaced by the NMT promoter to generate the final iKO construct p5'*nmt*TRAD4 –hDHFR-TetO7HA$_2$NMTΔ.

Figure S1.

A **TRAD4 AD:**

ATGGACGCGAACAACATGAACCACATCCCGTACTACAACGAC
ATGAATAACAATATCACGAACTACGCGAACATGACGGGCATCC
AGAACCTCAACAATATGAACCCGATCATGATTGACCCTAACAA
CAACGCGGACTCTGTCGAGGAATTCTACGACGCGATCGACGG
CTGCAACGTCGTCGACACGAACTACATGGTCGGCACAAACAA
CATGATTGACCAGATCCACTTCAACGACGTCACGTCTAACGTC
GGCAACATGAACAACAACTGA

B

MATLAB code used to randomize the amino acid sequence of PFF0200c_7.3

```
% read in textfile
orig = textread('input.txt', '%c');

% creating integers whose values are the length of input sequence
N = length(orig); M = length(orig);

% create array for randomized input sequence new = blanks(N);

% randomize input sequence for i = 1:N
X = ceil(rand*M); % generate a random number new(i) = orig(X);
% at position i in new, insert the
% residue at position X in orig orig(X) = [];
% remove base at position X from orig
M = length(orig); % reset length calculator used in
% random number generation step end

% output randomized sequence new
```

C **-TATi2 AD:**

LTLVPNWNNTQPYLGLFF

-TRAD1 AD:

TACHAPGWAQAPQGPLQEEEQAEGSDGLAAPLSPGQSCPPRGG
AVAGRVPVAGEKYAQWLDAIYTVCLKIDDLCAKLDPEFPNAMA
LWEQRGPGIDGARSLFVNGGCPGGYGSGGTSVSEAKETCWAEE
DPQKRKPCTEPQSVALHQTQQRRSLRSFAWCLCCHVLTSISNMH
ARDSTGRVDPTQRAFDNMNPGDASPPFGVMGEYSTAGSHCTG
MYSLGGGTPAHASTLPGSGAWAGGAGSLKKRGRK

-TRAD2 AD:

NNNNEHSESDKTECTYEVGQLQNIINNNLNVCNSNCNNANADN
NMFANNSNLLNSNEHNNIININKIDNQKYIDINDQNFDTIEDVTN
FDNISEKEDIDDERNSFITIDHENDKYMQWFDTIYTVCVKLDEL
CCKLNSSFPHSMNIWNQSGKVNMHHLKSVFNINENWIDIYNQD
CKKNKNDLLFDVNIQKNYNNPTCRDYNKNGMLNSNLSNDINN
SEFHYNIKDSTIIDNKNNLLSNPNNINNCVNYFPEKNNYSKVGI

-TRAD3 AD:

TSSAATSSSSVPPQLVVGDNTSNFGVCYGSNPNGGIYSHMSVMP
LRSDGSLCLMEALNRSSHSNHHQDSSPKVEDFFGTHHNNTSHK
EAMDLSLDSLFYNTTHEPNTTTNFQEFFSFPQTRNHEEETRNYG
NDPSLTHGGSFNVGVYGEFQQSLSLSMSPGSQSSCITGSHHHQQ
NQNQNHQSQNHQQISEALVETSVGFETTTMAAAKKKRGQEDV
VVVGQKQIVHRKS

-TRAD4:

DKNNMNHIPYYNDMNNNITNYANMTGIQNLNNMNPIMIDPNN
NADSVEEFYDAIDGCNVVDTNYMVGTNNMIDQIHFNDVTSNV
GNMNNN

-Ran-TRAD4 AD:

MHNNIANIVDNMCNNTVIDTLYDNDNDVMNMYTNYINNYFNT
MTVAYFVNNQINVDGSGPIINNMHNPINDMNNNDPQMEESGMH
ANNN

D

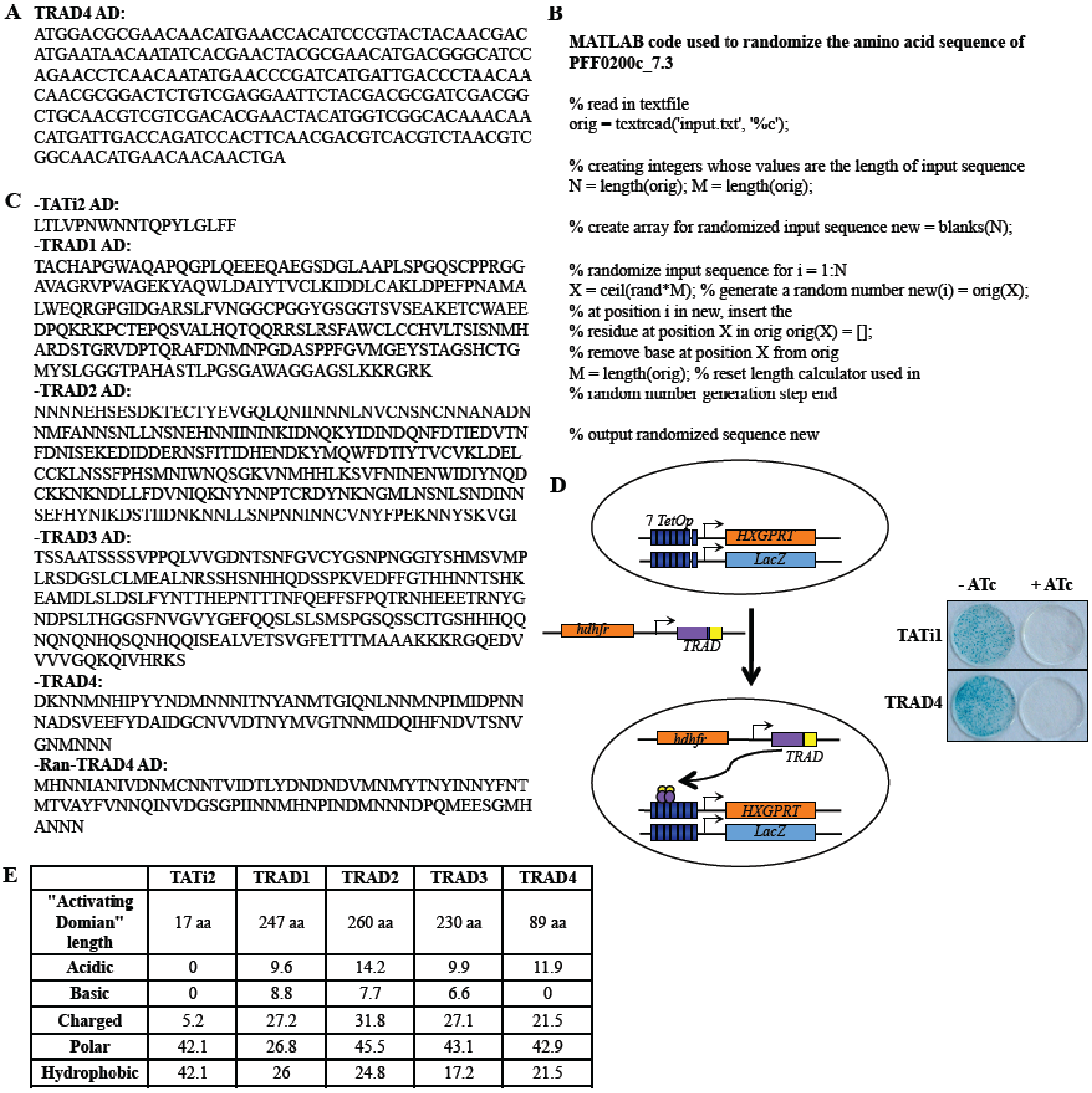

E

	TATi2	TRAD1	TRAD2	TRAD3	TRAD4
**"Activating Domian" length**	17 aa	247 aa	260 aa	230 aa	89 aa
**Acidic**	0	9.6	14.2	9.9	11.9
**Basic**	0	8.8	7.7	6.6	0
**Charged**	5.2	27.2	31.8	27.1	21.5
**Polar**	42.1	26.8	45.5	43.1	42.9
**Hydrophobic**	42.1	26	24.8	17.2	21.5

Figure S1. Related to Figure 1

(A) DNA sequence of the synthetic PfSIP2 activating domain used in the yeast two hybrid assays.

(B) MATLAB code used to randomize the amino acid sequence of PFF0200c_7.3.

(C) Amino acid sequences of putative transactivation domains.

(D) Scheme of the strategy used to validate activating domains. A recipient strain containing HXGPRT and LacZ under the control of a tet-transactivator responsive promoter (Meissner et al., 2001) was transfected with a linear DNA vector expressing fusion of the TetRep and various truncations of ApiAP2 transcription factors. The inset shows LacZ expression regulated by TATi-1 and TRAD4 in an ATc-dependent manner as determined by X-Gal staining.

(E) Amino acid composition of the activating domains. The percentages of acidic (DE), basic (KR), charged (RKHYCDE), polar (NCQSTY), and hydrophobic (AILFWY) amino acids in the transactivation domains were determined.

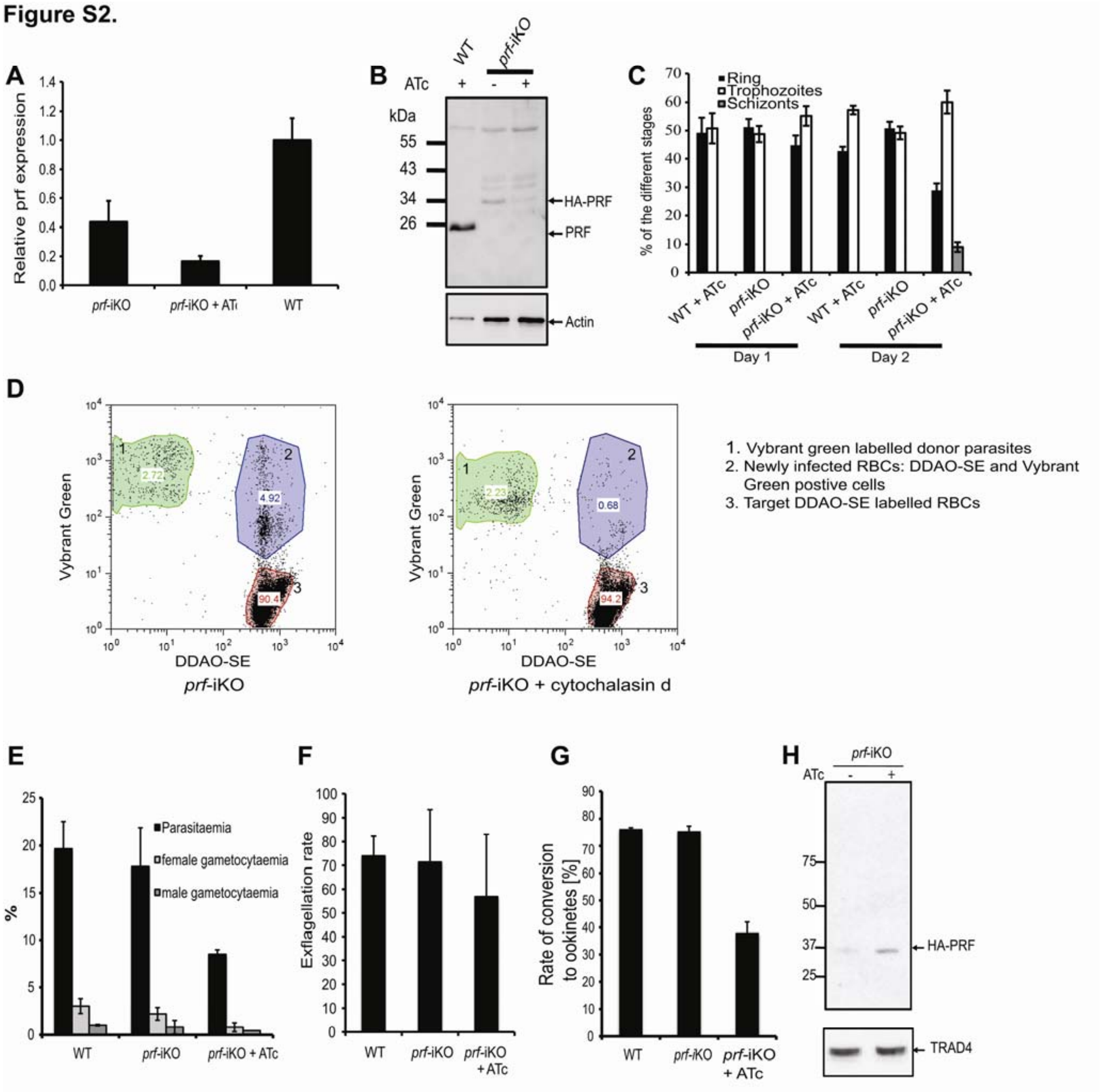

Figure S2. Analysis of *prf*-iKO parasites, Related to Figure 3

(A), (B) Impact of ATc on transcript and protein levels.

Relative transcript levels in mixed peripheral blood stages as determined by quantitative PCR. Mice were bled after 36 h of ATc in the drinking water where indicated. Quantitative PCR results from cDNA were normalized to PbH2A (PBANKA_111700) and 60S ribosomal protein L38e (PBANKA_091810) mRNA expression. Data are represented as the mean ± SD of three independent experiments.

(B) Western blot analysis of protein extracts from the same biological samples as above.

(C) Effect of *prf* knockdown on the stage composition of *P. berghei* in peripheral blood.

Percentage of rings, trophozoites and schizonts present *in vivo* was determined after 24 h, and 48 h of ATc treatment. At day one, the distribution of the different stages is similar in WT, prf-iKO and prf-iKO treated with ATc parasites. At day two, we observed a drop in the percentage of ring forms, an

increase in trophozoites and the appearance of schizonts suggesting a default in egress and/or invasion. Data are represented as the mean ± SD of three independent experiments.

(D) Invasion phenotype.

Target RBCs were labelled with the amine-reactive fluorescent dye DDAO-SE and analysed by FACS. Purified schizonts were labelled with the DNA dye Vybrant Green. Labelled target RBCs and schizonts were mixed under vigorous shaking for 20 minutes to allow invasion and analysed by FACS. All the cells containing labelled DNA were gated on FL1 and quantified; Cells labelling for both DDAO-SE and Vybrant Green are newly invaded RBCs. A representative experiment using *prf*-iKO parasites treated or not with cytochalasin D is shown.

(E) Effect of PRF down-regulation on gametocytogenesis following ATc treatment for 48 h. Gametocytemia was quantified by microscopic examination of Giemsa-stained blood smears. Data are represented as the mean ± SD of three independent experiments.

(F) Effect of PRF down-regulation on exflagellation. Data are represented as the mean ± SD of three independent experiments.

(G) Effect of ATc on macrogamete-to-ookinete conversion rates. Error bars show standard deviations of 4 replicates from two independent experiments.

(H) Western blot showing ATc induced reduction of HA-PRF in *prf*-iKO ookinetes. TRAD4-Ty expression served as loading control. ATc was administered in the drinking water for 48 h before gametocytes were differentiated into ookinetes *in vitro* in the continued presence of ATc and purified. Data are representative of two independent experiments.

Figure S3.

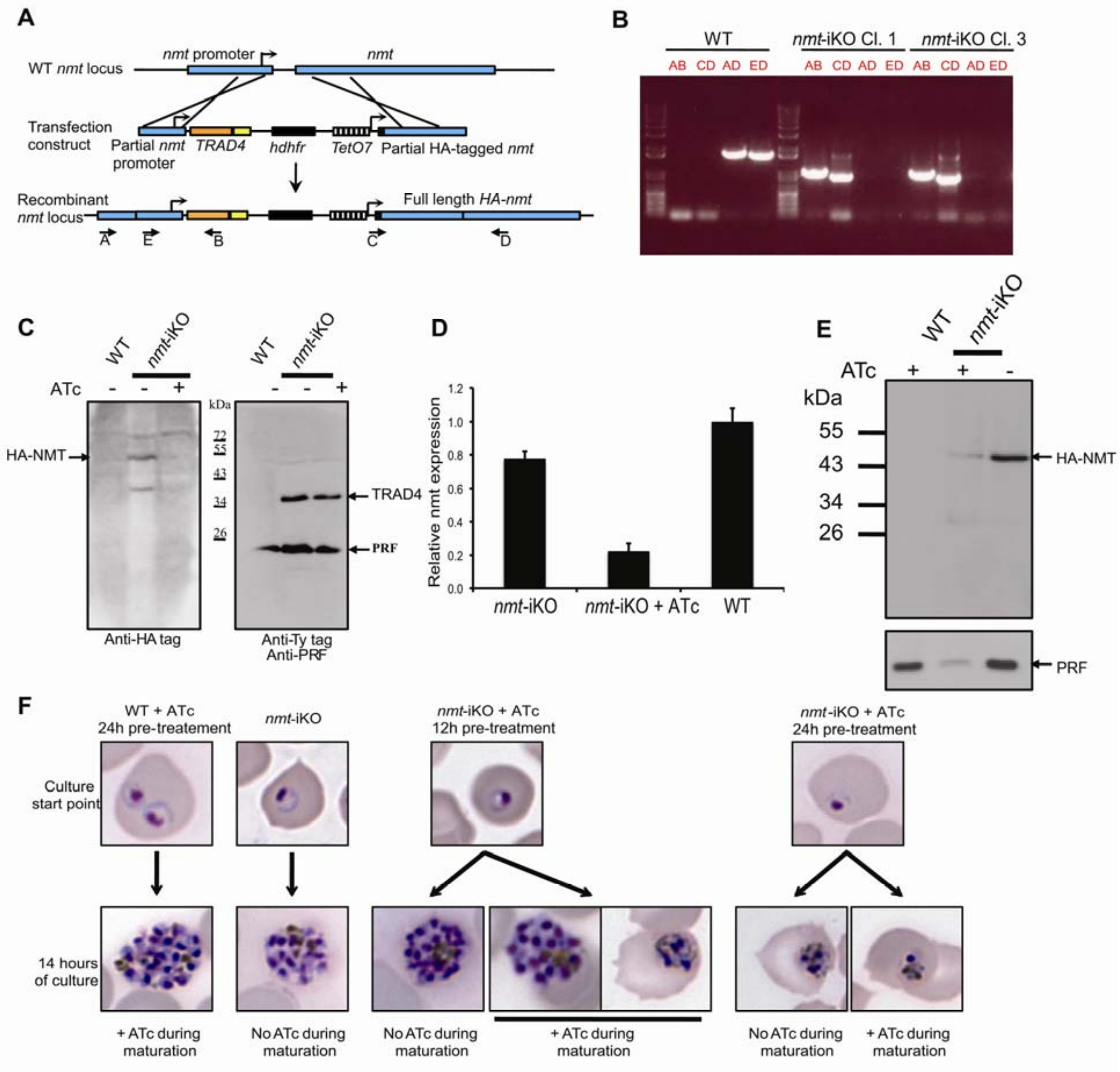

Figure S3. Related to Figure 4

(A) Schematic of the *nmt* transfection vector and recombinant locus.

(B) Agarose gel of genotyping PCR reactions performed on genomic DNA extracted from two parasite clones. Annealing sites of primer pairs (red) are shown in (A).

(C) Western blots analysis of protein extracts from mixed blood stages.

(D), Relative *nmt* transcript levels in mixed peripheral blood stages as determined by quantitative PCR. Mice were bled after 36 h of ATc in the drinking water where indicated. Quantitative PCR results from cDNA were normalized to PbH2A (PBANKA_111700) and 60S ribosomal protein L38e (PBANKA_091810) mRNA expression. Data are represented as the mean ± SD of three independent experiments.

(E) Western blot analysis of protein extracts from the same biological samples as in (D).

(F) Micrographs of Giemsa stained blood films prepared from infected mice. The top row shows representative parasites from mice that had been treated or not with ATc for 12 or 24 h. The bottom row shows representative schizonts 14 h later after parasites had been cultured *in vitro* under the conditions indicated.

Table S1. Primers used in the study, Related to the Experimental Procedures

Primers used for protein dissection.

Section	*Forward Primer 5' to 3'*	*Reverse Primer 5' to 3'*
PF14_0633_1	GCTACTGAATTCATGGAAGATAACAATATAATGAAC	GCAGGATCCTAAATTATTATGAGCATTCATACC
PF14_0633_2	GCTACTGAATTCGCATTACACAATAGTGATCTTG	GCAGGATCCTATATTCATGTTCATATTATAATTTTG
PF14_0633_3	GCTACTGAATTCGATGTAGAAAACTTGATGAATGTG	GCAGGATCCTAAATTTTTCCCATCTTTCATATTATT
PF14_0633_4	GCTACTGAATTCCCAAATATAATGGATATGGTTC	GCAGGATCCTAATAAATTGGAATCTCCTAAGTT
PF14_0633_5	GCTACTGAATTCACGCCTTTAGATAATTCTAATGG	GCAGGATCCAGATTCAGAATTATCATATGAATTT
PF11_0442_1	GCTACTGAATTCATGAATGAAACATTATTACATAATAA	GCAGTCGACATCTACAACATTTTTATCACTTTC
PF11_0442_2	GCTACTGAATTCACAAAAATAGACAACATAAATTTTG	GCAGTCGACTTGAGAAAAGGATGTTGGTTCATT
PF11_0442_3	GCTACTGAATTCGATAACGAAAATGTGAATGCTTC	GCAGTCGACATTTTTTATATTTTTCATATTTTTCATATT
PF11_0442_4	GCTACTGAATTCGATATGGAAAATATAGTGAAAAAG	GCAGTCGACATTGTTTATATGGTTACTTTGGTTT
PF11_0442_5	GCTACTGAATTCATATATCACATTCCTAGTTCTAATC	GCAGTCGACATCATCCATATGATTCATATTATATT
PF11_0442_6	GCTACTGAATTCAGTAACATATCAATGTATACTAAAA	GCAGTCGACATTTTGATGAAAAAATAAATCATTAG
PF11_0442_7	GCTACTGAATTCGGATTTAATAATTGTTCTTTATACAA	GCAGTCGACCGCATCTGTTTTGTTATTTTTATT
PfSIP2_1	GCTACTGAATTCATGGAAGATAATTTAGTTAAAGAATC	GCAGTCGACGTGTTCTCTCATTTTGTTTTGC
PfSIP2_2	GCTACTGAATTCATAGGTAGCCAAGAACCAGTC	GCAGTCGACTCCTTTAATTGAAAATGTTTTCAC
PfSIP2_3	GCTACTGAATTCGAAGTACCAAGTAGCACTTTG	GCAGTCGACTATATTATTATTTAATTGTGTCTTTAC
PfSIP2_4	GCTACTGAATTCAAAAATAGTTATTATATACCAAGTG	GCAGTCGACGTTACGATTTTTCGAAGATCTG
PfSIP2_5	GCTACTGAATTCACGTTAGAAACTCCATATGATAA	GCAGTCGACATTGTTCATGTTTACCATATTTGG
PfSIP2_6	GCTACTGAATTCTTAACAAATATAAATTCTGCGTATAA	GCAGTCGACTTCTAAGTCAATATAATAATTACAATT

PfSIP2_7	GCTACTGAATTCAATATGAATAGTACTAAGATTGATG	GCAGTCGACATTATTATTCATATTTCCTACATTAC
PfSIP2_8	GCTACTGAATTCCTAACCAATTTTAGTAATCCCTA	GCAGTCGACTATCATATCCGACCTATTAGATAA
PfSIP2_9	GCTACTGAATTCTCTTATAACATGTCTGGGTTAG	GCAGTCGACAGGCTCATTTTTTCTATTCTCTTTT
PfSIP2_10	GCTACTGAATTCTCATTTATTAATACTAATGTTAATGTT	GCAGTCGACTTTGTCTACATGTTCATTTATCTG
TATi-2	GCTACTGAATTCACTCTTGTTCCAAACTGGAAC	GCAGTCGACAAAGAATAGACCGAGATAGGG
TATi-3	GCTACTGAATTCTATCTCCTGCCAACGTGCATCCCTGTCGACGCA	TGCGTCGACAGGGATGCACGTTGGCAGGAGATAGAATTCAGTAGC

Primers used for subdivision of sections positive for activation.

Section	*Forward Primer 5' to 3'*	*Reverse Primer 5' to 3'*
PF14_0633_3.1	Same as PF14_0633_3_F	GCAGTCGACATTATTATTGTTATTGTTGTTGTTC
PF14_0633_3.2	GCTACTGAATTCAATAATAACAATAATAGTGGTAGTAA	GCAGTCGACGTTGTTATTATTTGAATCGTTATTT
PF14_0633_3.3	GCTACTGAATTCTCTAATTCTGGTAATAGCAATTC	GCAGTCGACTAAATTTTTCCCATCTTTCATATTATT
PF14_0633_4.1	GCTACTGAATTCAATAACAACCAATCAAATATATCTAA	GCAGTCGACTAATTCCATTTGCCCTTTAAGAC
PF14_0633_4.2	GCTACTGAATTCAATGATTTAAATTTTCGATTACATG	GCA**GTCGAC**ATTAGATTGGTTATAATAGACACAG
PF14_0633_4.3	GCTACTGAATTCAGCAAAAGAAAAAAAACTACATCT	GCAGTCGACTAATAAATTGGAATCTCCTAAGTT
PF11_0442_5.1	Same as PF11_0442_5_F	GCA**GTCGAC**ACTCAACCTACAACATAAATCATC
PF11_0442_5.2	GCTACTGAATTCAGGAAAGATTATATGAGGAATAG	GCAGTCGACAACAGACTTATTATTCCCTT
PF11_0442_5.3	GCTACTGAATTCGGTACATTTCCTCATTCAATGAA	Same as PF11_0442_5_R
PfSIP2_4.1	Same as PfSIP2_4_F	GCAGTCGACATTGTTATTATTATAAAGATAATAATAA
PfSIP2_4.2	GCTACTGAATTCGACAATGTAACTGATGAAGTCC	GCA**GTCGAC**ATCATAATTATTATTAATTCTTTTATC
PfSIP2_4.3	GCTACTGAATTCGGAATGTTAGTGAAAACATATGTAG	Same as PfSIP2_4_R
PfSIP2_6.1	Same as PfSIP2_6_F	GCA**GTCGAC**CCCTTTATTTACATTCCATCCAA

PfSIP2_6.2	GCTACTGAATTCGAATATCAATCACAAAGATTTTATG	GCAGTCGACTTTATTATTCATAATATTCTCTTTCTC
PfSIP2_6.3	GCTACTGAATTCGATGGTATGGAGAATAATAATATC	Same as PfSIP2_6_R
PfSIP2_7.1	Same as PfSIP2_7_F	GCAGTCGACTTTATCTATTATATTATGTACATCATT
PfSIP2_7.2	GCTACTGAATTCGATAATATTTCAAATTATAATAATCC	GCA**GTCGAC**AGAATCAGCATTATTATTTGGATC
PfSIP2_7.3	GCTACTGAATTCAATAATATGAACCATATACCATATT	Same as PfSIP2_7_R

Primers used to generate the *T. gondii* transfection plasmids

Primer names	*Forward Primer 5' to 3'*	*Reverse Primer 5' to 3'*
TRAD1 AD	CCTGCAGGAAGTGCACACGAATCAAGACATGCATCCTTTGGACACTGCATGCCATGCTCCAGGCTGG (TY tag included)	TTAATTAATCCTTTCGCCCCCGCTTCTTC
TRAD2 AD	ATGCATAATAACAACAATGAACATAGTGAAAGTGATAAAACGG	TTAATTAATTTATTCCAACTTTAGAATAGTTATTTTTTTCTGG
TRAD3 AD	ATGCATATGGATCTTAGCTTAGATAGTTTATTCTAC	TTAATTAAGATTTTCTATGAACAATCTGTTTCTGACC
TRAD4 randomized AD	CCGCTGCAGGAATTCAACAACATTGCTAATATAGTTGATAATATG	GGCTTAATTAATCGACACCATTATTATTTGCATGCATTCCAG

Primers used to generate the *P. berghei i*mCherry transfection plasmids

Section	*Forward Primer 5' to 3'*	*Reverse Primer 5' to 3'*
TetO7- mCherry	ATATCCGCGGATACTCGAGTTTACCACTCCCTATCAGT	GAGAGTCGACTTAAAATAAATTAAATACAATTAATG
PbEF1α promoter	ATATCCGCGGAGCTTAATTCTTTTCGAGCTC	GAGACTCGAGTTTTATAAAATTTTTTATTTATTTATAAGC
TRAD and 3'utr	ATATCTCGAGATGTCGCGCCTGGACAAGAG	ATATCTCGAGGAAATTGAAGGAAAAAACATCATTTG

Primers used to generate *prf* and *nmt* iKO constructs

Section	*Forward Primer 5' to 3'*	*Reverse Primer 5' to 3'*
prf promoter	GCCAAGCTTGCTAGCCGCGGCTTGTTTTAATTTGTTATTTTTTGTTG	GCCAAGCTTCCGCGGAGGGCTTAAAATATATTATATGAGGAAAGGG
prf coding	GCCGGCATGGAAGAATATTCATGGGAAAATTTTTTAAATGACAAAC	TTTGGCTAGCTATTATCAGAATAAAAAATTTTCACACACAC

TetO7-HA-PRF	GCCGTTAACTTTCGATACCGTCGACCTCGAG	CCGAGATCTGCGCTTTTTTTGTCCAAAATTTTAACGCTAGC
prf integration in 5’	CAAACATGGGAAATGGCAAAAGAAATGC	GAGCGAGTTTCCTTGTCGTCAGGCC
prf integration in 3’	CTGCAGCAAAATGTCAGGTTACCCCTATGACGTGC	ACTAGTTTATGCGGCACCTGTATCAGTGCTTTCG
nmt coding	GCCGCCGGCATGGATGGTGATAATGTAAGAAATAAATAAAAAAAAAATAAGAAG	CGGGCTAGCCAAGCATATCCTTCTGGTAGTTTGTACTCATC
nmt promoter	GGCCCGCGGGTACGTAAGAAGCATTACAACATTGCACG	CCGCCGCGGGATTTTTGTGATAGTTAAATATTTTTTTATAGATTTCTACAACC
nmt integration in 5’	ACCATAGCTACAATGATAAGCTATATGCAC	GAGCGAGTTTCCTTGTCGTCAGGCC
nmt integration in 3’	CTGCAGCAAAATGTCAGGTTACCCCTATGACGTGC	GTATCGACACGTTTTACTCCTATATGCC

Supplemental References

Janse, C.J., Franke-Fayard, B., Mair, G.R., Ramesar, J., Thiel, C., Engelmann, S., Matuschewski, K., van Gemert, G.J., Sauerwein, R.W., and Waters, A.P. (2006a). High efficiency transfection of *Plasmodium berghei* facilitates novel selection procedures. Mol Biochem Parasitol *145*, 60-70.

Janse, C.J., Ramesar, J., and Waters, A.P. (2006b). High-efficiency transfection and drug selection of genetically transformed blood stages of the rodent malaria parasite *Plasmodium berghei*. Nat Protoc *1*, 346-356.

Krizek, B.A., and Sulli, C. (2006). Mapping sequences required for nuclear localization and the transcriptional activation function of the *Arabidopsis* protein AINTEGUMENTA. Planta *224*, 612-621.

Meissner, M., Brecht, S., Bujard, H., and Soldati, D. (2001). Modulation of myosin A expression by a newly established tetracycline repressor-based inducible system in *Toxoplasma gondii*. Nucleic acids research *29*, E115.

Meissner, M., Krejany, E., Gilson, P.R., de Koning-Ward, T.F., Soldati, D., and Crabb, B.S. (2005). Tetracycline analogue-regulated transgene expression in *Plasmodium falciparum* blood stages using *Toxoplasma gondii* transactivators. Proceedings of the National Academy of Sciences of the United States of America *102*, 2980-2985.

Meissner, M., Schluter, D., and Soldati, D. (2002). Role of *Toxoplasma gondii* myosin A in powering parasite gliding and host cell invasion. Science *298*, 837-840.
